# Point-wise spatial network for identifying carcinoma at the upper digestive and respiratory tract

**DOI:** 10.1186/s12880-023-01076-5

**Published:** 2023-09-25

**Authors:** Lei Zhou, Huaili Jiang, Guangyao Li, Jiaye Ding, Cuicui Lv, Maoli Duan, Wenfeng Wang, Kongyang Chen, Na Shen, Xinsheng Huang

**Affiliations:** 1 Department of Otorhinolaryngology-Head and Neck Surgery, Zhongshan Hospital Affiliated to Fudan University, Xuhui District, 180 Fenglin Road, , Shanghai, 200032 P. R. China; 2 Department of Clinical Science, Intervention and Technology, Karolinska Institutet, Stockholm, Sweden; 3 Department of Otolaryngology Head and Neck Surgery, Karolinska University Hospital, 171 76 Stockholm, Sweden; 4 Institute of Artificial Intelligence and Blockchain, Guangzhou University, Guangzhou, 510006 P. R. China; 5 Pazhou Lab, Guangzhou, 510330 P. R. China

**Keywords:** Artificial intelligence, Oral pharynx, Hypopharynx, Larynx, Nasopharynx

## Abstract

**Problem:**

Artificial intelligence has been widely investigated for diagnosis and treatment strategy design, with some models proposed for detecting oral pharyngeal, nasopharyngeal, or laryngeal carcinoma. However, no comprehensive model has been established for these regions.

**Aim:**

Our hypothesis was that a common pattern in the cancerous appearance of these regions could be recognized and integrated into a single model, thus improving the efficacy of deep learning models.

**Methods:**

We utilized a point-wise spatial attention network model to perform semantic segmentation in these regions.

**Results:**

Our study demonstrated an excellent outcome, with an average mIoU of 86.3%, and an average pixel accuracy of 96.3%.

**Conclusion:**

The research confirmed that the mucosa of oral pharyngeal, nasopharyngeal, and laryngeal regions may share a common appearance, including the appearance of tumors, which can be recognized by a single artificial intelligence model. Therefore, a deep learning model could be constructed to effectively recognize these tumors.

## Introduction

In recent years, artificial intelligence (AI) technology has made significant strides in various fields of medicine, including the diagnosis of oral cancer [1, 2], dermatology disease [3], ocular fundus disease [4], lung cancer [5], pathological slices diagnosis [6] even the prediction of gene editing results [7]. AI is playing an increasingly important role in medicine, surpassing what was previously possible [8]. It may soon replace tedious or dangerous work with machines equipped with AI systems. Early detection of cancer has always been associated with a good prognosis. Therefore, detecting cancer in its early stages is crucial [1], regardless of whether it is done in the hospital or through self-diagnosis.

Head and Neck Squamous Cell Carcinoma (HNSC) is a common cancer worldwide [9]. Most HNSC cases occur in the mucosa of the nasopharynx, oral pharynx, hypopharynx, or larynx, which we defined as regional upper digestive and respiratory tract (rUDRT) here. Cancer in these regions can have a profound impact on patients' quality of life, causing dyspnea, dysphagia, and even voice loss. Early diagnosis and intervention can significantly improve patients' prognosis. Therefore, early and accurate detection of cancer in the rUDRT mucosa is crucial [10].

Several excellent AI diagnostic models have been developed to detect oral cancer [1, 2, 11–14]. However, few models have been used to diagnose cancer in rUDRT mucosa using a single model. Mohammed et al. reviewed the literature, and summarized the diagnosis of nasopharyngeal carcinoma; finding that most studies focus on predicting the prognosis of NPC using machine learning techniques [15]. They also constructed a deep learning model to detect NPC in microscopic image [16]. Endoscopic images-based deep learning model were also developed to detect nasopharyngeal carcinoma with good results [17, 18]. Uthoff et al. proposed an oral and oral pharyngeal cancer detection model, as well as a portable image collection tools to aid in self-diagnosis [14, 19]. Recently, Hao et al. established a deep learning model called DCNN to classify the tissues from normal, pre-cancerous, and benign ailments. The model had a sensitivity and specificity of 72.0% and 94.8%, respectively, and an area under curve (AUC) of 0.953[20]. Some benign disease diagnostic models have also been developed for mucosa diseases of the oral pharynx, such as the strep throat identification model [21], which can distinguish bacterial from viral infection of the throat. Van Staveren et al. constructed a diagnostic model for oral leukoplakia [22].

However, the machine learning method has rarely been applied in rUDRT using a single neural network model to identify cancers in all rUDRT mucosa. The model constructed to detect nasopharyngeal carcinoma was only used to identify nasopharyngeal cancer rather than laryngeal cancer, etc. Tumors in this region share common characteristics, such as irregular shape, ulceration, roughness, and tendency to bleed; while normal tissue has a smooth appearance and texture. These features suggest that the machine learning method may be suitable for identifying cancerous regions in an integrated AI model. This can facilitate self-monitoring of tumors in these regions, which may improve early tumor diagnosis. A new point-wise spatial attention network using semantic segmentation, was adopted to do the cancerous region detection [23]. Here we reported the detailed design and training process.

### Contributions:

1. The research in this article has confirmed that the mucosa of rUDRT shares a common appearance, as well as the tumor appearance which can be recognized by a single integrated deep learning neural network model.
2. The finding implies that an integrated AI model could be constructed to detect tumors in the rUDRT region.
3. The finding here facilitate a pan cancer detection deep learning neural network model, with the combination with portable self-examination equipments, this may facilitate the easily early diagnosis of the carcinoma in this region.

## Materials and methods

### Image data collection and data augmentation

To conduct this study, 1742 cancerous endoscopic images from 101 patients were collected and labeled by two experts in this field. These patients were all histologically proved squamous carcinoma. And 6473 normal or benign lesion images of rUDRT from 200 patients were prepared. Before model training, we also carried out data enhancement on the cancerous images through image rotation, scaling, shearing, panning, and image flipping, etc. The cancerous endoscopic images increased from 1742 to 8725.

During endoscopy, endoscopists always took many images of the tumor from different angles to achieve a comprehensive perception. As a result, one case may be taken several images from different positions, which was similar to the data augmentation process. As a result, these similar images were all adopted, labeled and used in the training, testing as well as validation process.

During machine learning, many algorithms and models have a very basic assumption that the data distribution is homogeneous. If we apply the algorithm directly to the above data, in most cases we will not achieve the desired result because the uneven distribution that the non-malignant images are several times more than malignant tumor. Therefore, we need to enhance the data for the cancerous images, so that the number of cancerous images is about the same or even more than the number of benign images, as the main objective of our model training is to identify the cancerous areas of the images. We eventually expanded the number of cancerous images to 8,725 by randomly flipping them and other common data enhancement methods.

We train the PSANet model for image segmentation of rUDRT medical images. The core idea of this model is to use the spatial attention mechanism to enhance the feature representation ability of CNN model at the pixel level, so as to achieve more accurate scene analysis results.

With adaptive predictive attention graphs, each position in the feature map is connected to all other positions to gather a variety of information near and far away. In addition, to fully understand the complex scene, we designed a bi-directional information propagation path. Each location collects information about all other locations to help predict itself, and vice versa, and then information from each location can be globally distributed to help predict all other locations. Finally, the bi-directional aggregated context information is fused with local features to form the final representation of complex scenes.

### Selection of models

In order to find a semantic segmentation model that performs well on the cancer region detection task, we have selected some classical semantic segmentation models for comparison experiments, such as Accuracy (Acc) and IOU. Acc can be understood as the percentage of pixels in an image that are correctly classified, and the class imbalance problem occurs when one or some classes dominate in the data, while some other classes are only a small part of the image. At this point, Acc is not able to evaluate the performance of the model very well, as a good performing model must be able to have a high accuracy rate for all classes trained. Therefore, this paper also introduces the evaluation metric IOU, which is simply the area of overlap between predicted segmentation and live annotation divided by the joint area between predicted segmentation and live annotation. The range of this metric is 0–1 (0–100%), with 0 indicating no overlap and 1 indicating a fully overlapping segmentation.

In Table 1, mAcc and mIoU are calculated separately for each category and then averaged by category. As shown in Table 1, The mIOU and mAcc in PSANet are 86.83 and 92.38, receptivity, outperforming than other models. The basic reason is that the PSANet model is more reasonable. By using the spatial relationship between points to enhance the ability of feature representation, PSANet has higher efficiency and accuracy than other existing methods.

**Table 1:** Model training results

Method	mIOU	mAcc
FCN-UNet	63.93	69.15
PSPNet	86.6	91.92
DeepLabv3	86.53	90.96
EMANet	80.69	88.24
PSANet	86.83	92.38

The main models involved in the comparison experiments are: FCN-UNet, PSPNet [24], DeepLabv3 [25], EMANet [26] and PSANet [23]. The FCN-UNet model combines the features of the very classical FCN [27] and UNet [28] in the development of semantic segmentation techniques, with the symmetric structure of UNet for the feature extraction part of the model and the structure of FCN for the decoupling head part. The PSPNet framework is mainly based on the FCN approach and provides a pyramid pooling module for fusing features at different levels to achieve a fusion of semantics and details. DeepLabv3 not only improves the ability of the model to capture contextual information through the Atrous Spatial Pyramid Pooling module, but also uses Conditional Random Field as a post-processing tool to make image boundary segmentation more accurate. EMANet [26] and PSANet are attention-based semantic segmentation models that improve the model's ability to capture global information by introducing a self-attentive mechanism. In this paper, we use MMSegmentation [29], an open source target detection framework from Shang Tang Technology, which basically includes the mainstream semantic segmentation algorithms. In order to ensure a fair comparison, we use the same dataset and pre-training weights, and set the same hyperparameters such as training times and input image sizes. The experimental results are shown in Table 1 below, from which it can be concluded that PSANet performs better for cancer region detection on rUDRT images.

Hence, a point-wise spatial attention network (PSANet) was adopted to address this study [23], which can aggregate long-range contextual information in a flexible and adaptive manner. This model was constructed by Zhao et al. at 2018, which achieved top performance on various competitive scenes parsing datasets, including ADE20K, PASCAL VOC 2012, and Cityscapes, demonstrating its effectiveness and generality [23]. The backbone of this model was ResNet [30], which was the champion in the competition ImageNet 2015. In this study transfer learning was used to detect the cancerous region of the rUDRT by using PSANet.

### Training process and environment setting

To test this model and validate our hypothesis, 8725 labeled tumor images and 6473 normal mucosa images of the rUDRT were prepared. These images were labeled by the Labelme (v4.2.9) software, which was used to tell the model which part of the image was the tumor region, where the masks have a value of 0 for pixels considered to be normal, and a value of 1 for pixels of being cancerous. The image dataset was constructed according to the VOC 2012 semantic segmentation format. Among all of the 15198 images, 80% were randomly selected for training and the rest 20% were randomly selected as validation and testing set, among which 1/3 was set as testing, the other 2/3 was set as validation set. The details of the split were listed in Table 2.

**Table 2:** The image split in this study

	Training	Testing	Validation	Total
Cancer	6979	583	1163	8725
Normal	5179	431	863	6473

All images were resized to 480 × 480 pixels. The color channel was converted to RGB and the pixel values of all the three channels were standardized to a floating-point number between 0 and 1. Then the pixel values are normalized by the following formulation (1).

1 $${p}_{input}=\frac{p-{p}_{mean}}{\partial }$$

where ${p}_{input}$ is the input pixel value to the PSANet, $p$ is the current pixel value,${p}_{mean}=\left[0.485, 0.456, 0.406\right]$, and $\partial =[0.229, 0 .224, 0 .225]$ [23]. At last the model was subject to training process. We have several model training parameter in the training process. For example, we use the Adam as the optimizer, where the learning rate is set to $2\times {10}^{-4}$, the decay rate is set to $6\times {10}^{-8}$, and the batch size is set to 8.

**Fig. 1:**
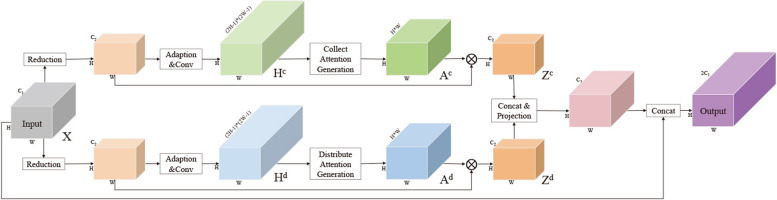
Architecture of the PSANet [23] used in this study

In order to improve the model training, we tried to add auxiliary losses to the original model structure of PSANet, and the training results are shown in Table 3. From the results, although the mIOU values decreased, our improvement did have the effect of improving the accuracy of the model, increasing from 92.38 to 96.3. The training was conducted with the PyTorch deep learning framework. The total number of epochs was 50, where the total number of iterations was 27450. The total training time was about 24 h on an Ubuntu 18.04 system by using an NVIDIA Tesla V100 (32 G memory). Figure 1 illustrated the architecture of the PSANet used in this study.

**Table 3:** Training results

Method	mIOU	mAcc
PSANet	86.83	92.38
PSANet + Auxiliary Loss	86.25	96.3

## Results

### Validation process

**Fig. 2:**
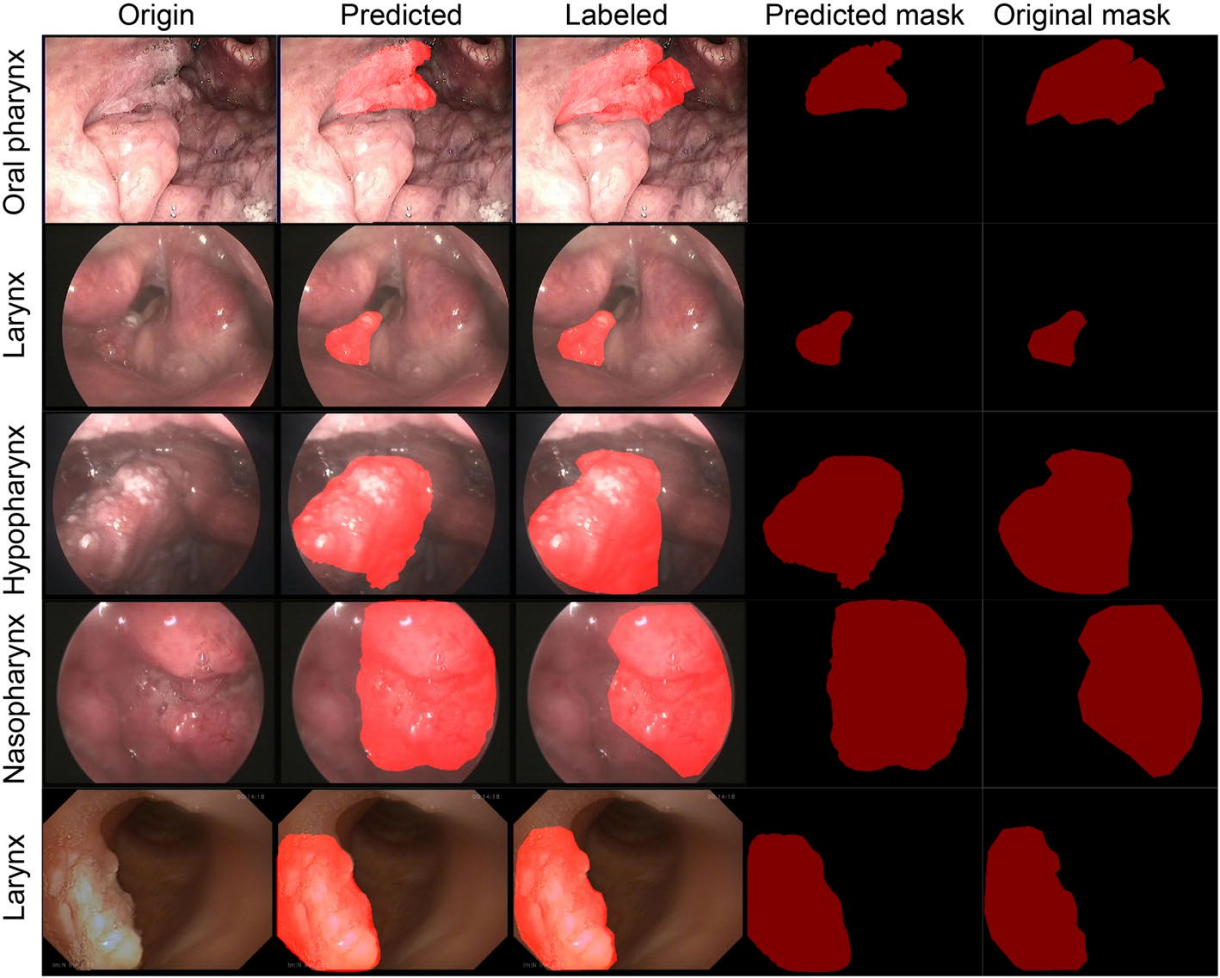
The predicted masks and comparison with the manually constructed masks with the Labelme software

After the training process, 2026 labeled validation images were loaded to validate the model’s accuracy. Figure 2 illustrated the predicted masks compared with the previous manually labeled actual masks by two experts in this field. The first column illustrated the original images derived from the endoscopy of rUDRT. The predicted masks overlaid on the top of the original images were illustrated in the second column. The third column showed the manually labeled mask overlaid on the top of the original images. The comparison of predicted masks with the manually labeled actual masks in the last two columns was made by two experts in this field.

Because there are many types of equipment in the clinical work, the image tone or color style may be different among each other. Figure 2. listed the three image sources, the first row images were captured by XION soft endoscope, the 2, 3, and 4 rows were captured by XION rigid endoscope, and the 5 row images were captured by AOHUA soft endoscope. All endoscopic images were captured from each patient under local anesthesia. Standard white light was used during image capture. All the three source images were pulled in the training process to enhance the robustness of the model [20].

**Fig. 3:**
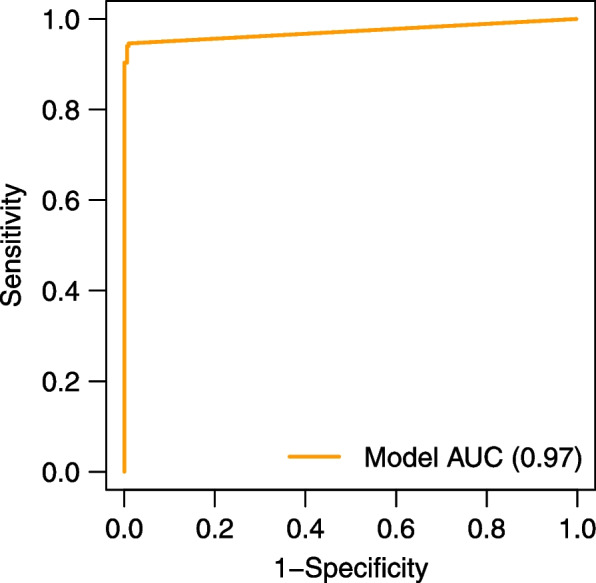
The ROC curve of the PASNet model used in this study

The accuracy of the proposed model in performing rUDRT cancer detection was evaluated using two criteria, namely the sensitivity as in Eq. (2) and the specificity as in Eq. (3). The semantic segmentation was evaluated by mIoU as in Eq. (4) and average pixel accuracy.

2 $$Sensitivity=\frac{TP}{TP+FN}$$

3 $$Specificity=\frac{TN}{TN+FP}$$

4 $$mIoU= \frac{{mask}_{segmentation}\cap {mask}_{truth}}{{mask}_{segmentation}\cup {mask}_{truth}}$$

where $\mathrm{TP}$ denoted true positive, $\mathrm{TN}$ denoted true negative, and $\mathrm{FP}$ and $\mathrm{FN}$ denoted false positive and false negative, respectively. The ${mask}_{segmentation}$ denoted the mask predicted by model and the ${mask}_{truth}$ was the true mask. The calculated sensitivity was 94.39% and the specificity was 98.68%. Figure 3 is the receiver operating characteristic curve (ROC), indicted that the AUC is 0.97. The calculated average mIoU was 86.25%, and the average pixel accuracy was 96.3%. The true positive or negative and predicted positive or negative data was listed in Table 4.

**Table 4:** The false positive and false negative images

	True positive	True negative
Predicted positive	202	2
Predicted negative	12	150

### Additional validation

**Fig. 4:**
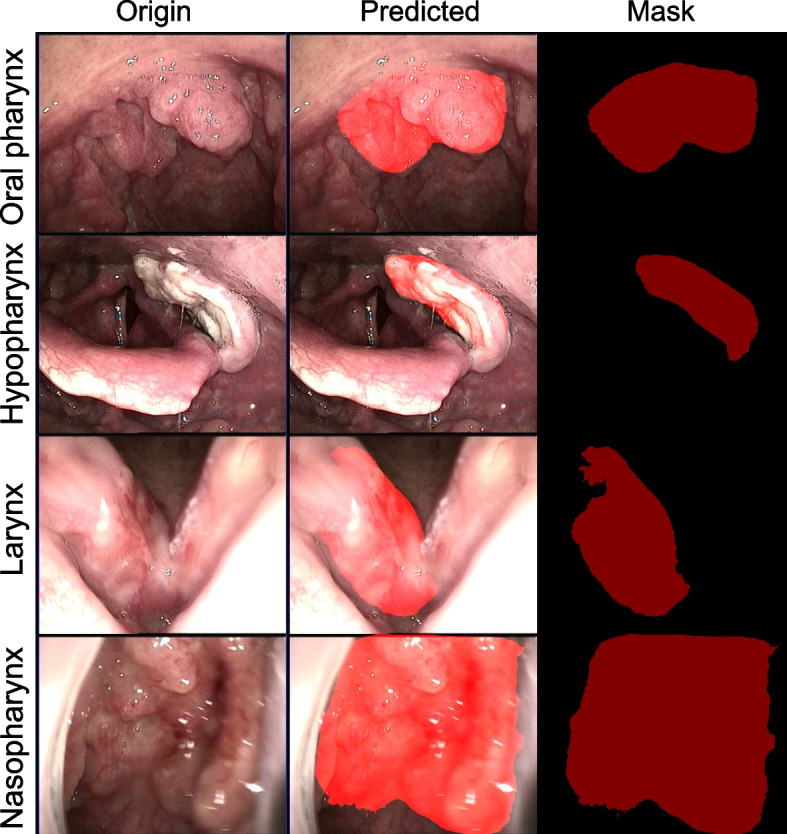
The predicted carcinoma region masks of the rUDRT carcinoma using thePSANet

After training and validation, in order to guarantee the independency between the training set and the testing set, other new endoscopic rUDRT images from clinically proven carcinoma were loaded to do the validation step. The prediction results were illustrated in Fig. 4. showing a well match with the cancerous contours labeled by experts in this field. The first column contained the original images; the second column illustrated the predicted masks overlaid on the top of the original images. And the third column was the masks predicted by this model.

## Discussion

### The images choosing and labeling

Medical image research using machine learning methods often suffers from a lack of training images. To address this, various augmentation strategies such as rotation and cropping have been used in the training of deep neural networks [20]. In this study, during examination, endoscopists took multiple images from different positions of a carcinoma case, which were selected except for the blurred ones. The goal of the study was to recognize the carcinoma as the region of interest (ROI), so the precise cancerous region was labeled. Images from at least three different endoscopes were chosen to capture images of enrolled patients, resulting in training images with varying tone and size, which could enhance the model's robustness [20].

### The prediction accuracy

The prediction accuracy was evaluated using four metrics: sensitivity, specificity, average mIoU, and average pixel accuracy. Xiong et al. reported a sensitivity of 72.0%, specificity of 94.8%, and an AUC of 95.3% in detecting laryngeal cancer from pre-cancerous lesions, [20]. Li et al. constructed a deep learning neural network to recognize the nasopharyngeal carcinoma, achieving a sensitivity of 91.3%, specificity of 83.1%, and an overall accuracy of 88.7% [17]. In the current study, the sensitivity was 94.4% and specificity was 98.7%, while the mIoU score was 86.3%, and the average pixel accuracy was 96.3%. The PSANet used in this study effectively aggregated information with global attention maps, capturing long-range contextual information effectively and improve scene parsing performance [23]. The results demonstrate the key role of context information for image understanding [23].

### Limitations

Limitations of the study include the collection of images from a single tertiary care center, insufficient diversity and number of endoscopic images, and the lack of classification from other precancerous lesions. Future studies should collect and analyze multicenter images and provide classification processes, such as differentiating cancer from benign tumors or infections like polyps, cysts, or edema. Additionally, finding a combination method of the AI model with a portable self-diagnosis device would be beneficial.

### The implication to the model design and application

The transfer learning method of retraining a previously trained deep neural network model with the endoscope images of rUDRT regions proved effective in this study. The PSANet model was able to recognize cancerous regions of rUDRT carcinoma, suggesting that an integrated AI model can be constructed to detect tumors in these regions. Previous models were designed to recognize different regions separately. However, this study demonstrated that carcinomas in these regions share common visual characteristics that could be utilized to design an integrated AI model, reanimating huge expectations for future applications. This pan-cancer detection model, combined with portable self-examination equipment [19], could facilitate the early diagnosis of carcinomas in the rUDRT region.

## Conclusion

This research confirms that the mucosa of rUDRT has a common appearance, including the appearance of tumors, which can be recognized by a single deep learning neural network model. This suggests that an integrated AI model could be designed to detect tumors in these regions, leading to the development of a pan-cancer detection deep learning neural network model in the future. This could also extend to other mucosa cancer.

In practice, this could facilitate the self-monitor of tumors in these regions, improving the early detection of tumors. Portable image collection tools aid in self-diagnosis [14, 19]. With the development of a pan-cancer detection model, monitoring of mucosa cancer in the rUDRT region could be improved. The advancement of portable video laryngoscopes may also facilitate self-diagnosis in the future. All of these developments hold promise for improving the early diagnosis of the rUDRT cancer.

## Data Availability

The datasets used and/or analysed during the current study available from the corresponding author on reasonable request.

## References

[1] Lu J, Sladoje N, Runow Stark C, Darai Ramqvist E, Hirsch J, Lindblad J. A deep learning based pipeline for efficient oral cancer screening on whole slide images. Image Analysis Recognition. 2020. p. 249.

[2] Bhandari B, Alsadoon A, Prasad PWC, Abdullah S, Haddad S (2020). Deep learning neural network for texture feature extraction in oral cancer: enhanced loss function. Multimedia Tools Appl.

[3] Tschandl P, Codella N, Akay B, Argenziano G, Braun R, Cabo H, Gutman D, Halpern A, Helba B, Hofmann-Wellenhof R, Lallas A, Lapins J, Longo C, Malvehy J, Marchetti M, Marghoob A, Menzies S, Oakley A, Paoli J, Puig S, Rinner C, Rosendahl C, Scope A, Sinz C, Soyer H, Thomas L, Zalaudek I, Kittler H (2019). Comparison of the accuracy of human readers versus machine-learning algorithms for pigmented skin lesion classification: an open, web-based, international, diagnostic study. Lancet Oncol.

[4] Varadarajan A, Poplin R, Blumer K, Angermueller C, Ledsam J, Chopra R, Keane P, Corrado G, Peng L, Webster D (2018). Deep learning for predicting refractive error from retinal fundus images. Invest Ophthalmol Vis Sci.

[5] Ardila D, Kiraly A, Bharadwaj S, Choi B, Reicher J, Peng L, Tse D, Etemadi M, Ye W, Corrado G, Naidich D, Shetty S (2019). End-to-end lung cancer screening with three-dimensional deep learning on low-dose chest computed tomography. Nat Med.

[6] Niazi M, Parwani A, Gurcan M (2019). Digital pathology and artificial intelligence. Lancet Oncol.

[7] Leenay R, Aghazadeh A, Hiatt J, Tse D, Roth T, Apathy R, Shifrut E, Hultquist J, Krogan N, Wu Z, Cirolia G, Canaj H, Leonetti M, Marson A, May A, Zou J (2019). Large dataset enables prediction of repair after CRISPR-Cas9 editing in primary T cells. Nat Biotechnol.

[8] Rajkomar A, Dean J, Kohane I (2019). Machine Learning in Medicine. N Engl J Med.

[9] Siegel R, Miller K, Jemal A (2019). Cancer statistics, 2019. CA Cancer J Clin.

[10] Mohammed MA, Ghani MKA, Hamed RI, Ibrahim DA (2017). Analysis of an electronic methods for nasopharyngeal carcinoma: Prevalence, diagnosis, challenges and technologies. J Comput Sci.

[11] Chan C, Huang T, Chen C, Lee C, Chan M, Chung P (2019). Texture-map-based branch-collaborative network for oral cancer detection. IEEE Trans Biomed Circuits Syst.

[12] Heidari A, Pham T, Ifegwu I, Burwell R, Armstrong W, Tjoson T, Whyte S, Giorgioni C, Wang B, Wong B, Chen Z (2019). The use of optical coherence tomography and convolutional neural networks to distinguish normal and abnormal oral mucosa. J Biophotonics.

[13] Jeyaraj P, Samuel NE (2019). Computer-assisted medical image classification for early diagnosis of oral cancer employing deep learning algorithm. J Cancer Res Clin Oncol.

[14] Uthoff R, Song B, Sunny S, Patrick S, Suresh A, Kolur T, Keerthi G, Spires O, Anbarani A, Wilder-Smith P, Kuriakose M, Birur P, Liang R (2018). Point-of-care, smartphone-based, dual-modality, dual-view, oral cancer screening device with neural network classification for low-resource communities. PLoS One.

[15] Mohammed MA, Abd Ghani MK, Hamed RI, Ibrahim DA (2017). Review on nasopharyngeal carcinoma: concepts, methods of analysis, segmentation, classification, prediction and impact: a review of the research literature. J Comput Sci.

[16] Mohammed MA, Abd Ghani MK, Hamed RI, Ibrahim DA, Abdullah MK (2017). Artificial neural networks for automatic segmentation and identification of nasopharyngeal carcinoma. J Comput Sci.

[17] Li C, Jing B, Ke L, Li B, Xia W, He C, Qian C, Zhao C, Mai H, Chen M (2018). Development and validation of an endoscopic images-based deep learning model for detection with nasopharyngeal malignancies. Cancer Commun.

[18] Abd Ghani MK, Mohammed MA, Arunkumar N, Mostafa SA, Ibrahim DA, Abdullah MK, Jaber MM, Abdulhay E, Ramirez-Gonzalez G, Burhanuddin MA (2020). Decision-level fusion scheme for nasopharyngeal carcinoma identification using machine learning techniques. Neural Comput Appl.

[19] Uthoff R, Song B, Sunny S, Patrick S, Suresh A, Kolur T, Gurushanth K, Wooten K, Gupta V, Platek M, Singh A, Wilder-Smith P, Kuriakose M, Birur P, Liang R (2019). Small form factor, flexible, dual-modality handheld probe for smartphone-based, point-of-care oral and oropharyngeal cancer screening. J Biomed Opt.

[20] Xiong H, Lin P, Yu J, Ye J, Xiao L, Tao Y, Jiang Z, Lin W, Liu M, Xu J, Hu W, Lu Y, Liu H, Li Y, Zheng Y, Yang H (2019). Computer-aided diagnosis of laryngeal cancer via deep learning based on laryngoscopic images. EBioMedicine.

[21] Askarian B, Yoo S, Chong J (2019). Novel image processing method for detecting strep throat (streptococcal pharyngitis) using smartphone. Sensors.

[22] Van Staveren H, Van Veen R, Speelman O, Witjes M, Star W, Roodenburg J (2000). Classification of clinical autofluorescence spectra of oral leukoplakia using an artificial neural network: a pilot study. Oral Oncol.

[23] Zhao H, Zhang Y, Liu S, Shi J, Loy CC, Lin D, Jia J. PSANet: Point-wise spatial attention network for scene parsing. European Conference on Computer Vision. 2018. p. 270.

[24] Hengshuang Z, Jianping S, Xiaojuan Q, Xiaogang W, Jiaya J. Pyramid scene parsing network. In: 2017 IEEE Conference on Computer Vision and Pattern Recognition (CVPR). 2017. p. 6230.

[25] Chen L, Papandreou G, Schroff F, Adam H. Rethinking atrous convolution for semantic image segmentation. 2017.

[26] Li X, Zhong Z, Wu J, Yang Y, Liu H. Expectation-maximization attention networks for semantic segmentation. In: 2019 IEEE/CVF International Conference on Computer Vision (ICCV). 2019

[27] Dai J, Li Y, He K, Sun J. R-FCN: object detection via region-based fully convolutional networks. In: Advances in Neural Information Processing Systems, vol. 2016

[28] Ronneberger O, Fischer P, Brox T. U-Net: Convolutional Networks for Biomedical Image Segmentation. CoRR. abs/1505.04597. 2015.

[29] MMSegmentation: OpenMMLab semantic segmentation toolbox and Benchmark. https://github.com/open-mmlab/mmsegmentation.

[30] He K, Zhang X, Ren S, Sun J. Deep residual learning for image recognition. 2016 IEEE Conference on Computer Vision and Pattern Recognition (CVPR). 2016. p. 770.

